# High versus Moderate Intensity Running Exercise to Impact Cardiometabolic Risk Factors: The Randomized Controlled RUSH-Study

**DOI:** 10.1155/2014/843095

**Published:** 2014-03-11

**Authors:** Wolfgang Kemmler, Michael Scharf, Michael Lell, Carina Petrasek, Simon von Stengel

**Affiliations:** ^1^Institute of Medical Physics, Friedrich-Alexander University Erlangen-Nürnberg, Henkestrasse 91, 91052 Erlangen, Germany; ^2^Institute of Radiology, Friedrich-Alexander University Erlangen-Nürnberg, Maximiliansplatz 1, 91054 Erlangen, Germany

## Abstract

Aerobic exercise positively impacts cardiometabolic risk factors and diseases; however, the most effective exercise training strategies have yet to be identified. To determine the effect of high intensity (interval) training (HI(I)T) versus moderate intensity continuous exercise (MICE) training on cardiometabolic risk factors and cardiorespiratory fitness we conducted a 16-week crossover RCT with partial blinding. Eighty-one healthy untrained middle-aged males were randomly assigned to two study arms: (1) a HI(I)T-group and (2) a sedentary control/MICE-group that started their MICE protocol after their control status. HI(I)T focused on interval training (90 sec to 12 min >85–97.5% HRmax) intermitted by active recovery (1–3 min at 65–70% HRmax), while MICE consisted of continuous running at 65–75% HRmax. Both exercise groups progressively performed 2–4 running sessions/week of 35 to 90 min/session; however, protocols were adjusted to attain similar total work (i.e., isocaloric conditions). With respect to cardiometabolic risk factors and cardiorespiratory fitness both exercise groups demonstrated similar significant positive effects on MetS-*Z*-Score (HI(I)T: −2.06 ± 1.31, *P* = .001 versus MICE: −1.60 ± 1.77, *P* = .001) and (relative) VO_2_max (HI(I)T: 15.6 ± 9.3%, *P* = .001 versus MICE: 10.6 ± 9.6%, *P* = .001) compared with the sedentary control group. In conclusion, both exercise programs were comparably effective for improving cardiometabolic indices and cardiorespiratory fitness in untrained middle-aged males.

## 1. Introduction

Higher levels of cardiorespiratory fitness are associated with lower risk of all causes of mortality and cardiovascular-/coronary heart diseases (CVD/CHD) [1]. Exercise significantly impacts cardiorespiratory fitness and is thus strongly recommended in both primary and secondary prevention of cardiometabolic diseases [2–4]. However, the most efficient exercise training strategies to positively affect cardiorespiratory fitness and cardiometabolic risk factors have yet to be identified. With respect to exercise intensity, there is some evidence that walking may be as effective as running for reducing cardiometabolic risk [5] at least when adjusting for energy expenditure (i.e., work). However, running is much more time efficient due to its higher physiological and biomechanical intensity [6]. This aspect is substantial, since lack of time was consistently reported as one of the central reasons for inactivity in Germany [7]. Developing this idea further, low volume/high intensity protocols should be a promising tool for impacting cardiometabolic risk in sedentary subjects. In fact, a number of corresponding studies that focus on prevention or rehabilitation of metabolic and cardiac diseases including myocardial infarction [8] applied low volume/high intensity (interval) training (“HI(I)T”) [9–13]. Beside its apparent time effectiveness a main pro for HIIT is its significantly higher effect on VO_2_max, the parameter considered as the typical marker of cardiorespiratory fitness [14], compared with “moderate intensity continuous exercise” (MICE) protocols traditionally applied in this research field (review in [15–19]). Since most of these studies focus on subjects with severe CAD and/or heart failure (review in: [15–17, 19, 20]) exercising on electronically braked cycle ergometers, it is doubtful, whether these data are transferable to the general population. Comparative studies that address primarily prevention of CVD/CHD are scarce [9, 13, 21–24].

For that reason, the primary purpose of this randomized controlled trial is to compare the effects of two running exercise protocols (HIIT versus MICE) under the premise of comparable “energy consumption” on the Metabolic Syndrome (MetS) as a sensitive cluster of metabolic and cardiac risk factors in untrained middle-aged males.

The primary hypothesis was that HIIT is significantly superior for impacting the Metabolic Syndrome (MetS) *Z*-Score and the number of criteria of the MetS in this cohort. The secondary hypothesis was that HIIT is significantly more effective for increasing cardiorespiratory fitness compared with MICE.

## 2. Materials and Methods

**Figure 1:**
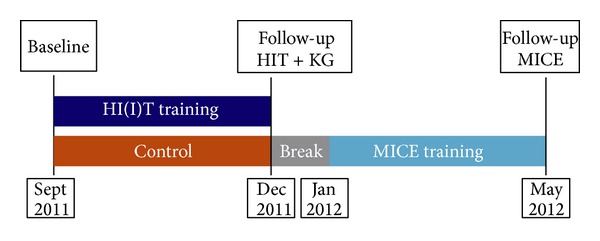
Time chart of the RUSH study.

The Running Study and Heart (RUSH) Trial was a 16-week randomized controlled crossover study with three study arms (two exercise, one waiting/control group) of untrained middle-aged males (Figure 1). The study was conducted by the Institute of Medical Physics, Friedrich-Alexander University Erlangen-Nürnberg (FAU), Germany, during April 2011 and July 2012. The primary study aim was to compare the effects of a high intensity (interval) training protocol (HI(I)T) versus a moderate intensity continuous exercise protocol (MICE) on physical performance, metabolic, and cardiac parameters. The study protocol was approved by the ethics committee of the Friedrich-Alexander University (FAU) of Erlangen-Nürnberg (Ethikantrag 4463). All the study participants gave written informed consent. The study was registered under www.clinicaltrials.gov (NCT01406730).

### 2.1. Main Endpoints

The primary study endpoints of the present contribution were as follows.

- Metabolic Syndrome (MetS) *Z*-Score according to Johnson et al. [25].
- Number of MetS parameters according to the National Cholesterol Education Programme Adult Treatment Panel III (NCEP ATP III) definition [26].

Secondary study endpoints were as follows.

- Maximum aerobic capacity (VO_2_max)
- Metabolic Syndrome criteria constituting the NCEP ATP III MetS criteria (i.e., waist circumference, mean arterial pressure (MAP), fasting glucose, triglycerides, and HDL-C).

### 2.2. Study Participants, Inclusion, and Exclusion Criteria

Study characteristic procedures of recruitment and the flow chart of the trial were described in more details in an earlier publication [27]. In summary (Figure 4), detailed announcements in local newspapers or on radio stations addressed untrained male subjects 30–50 years old. 121 subjects responded and were assessed for eligibility. Of these, 24 subjects had to be excluded due to the criteria of (a) male, 30–50 years old (*n* = 3), (b) “untrained” (i.e., ≤1 endurance exercise session/week; ≤2 total exercise sessions/week during the last 2 years; *n* = 12), (c) inflammatory diseases and pathological changes of the heart (*n* = 3), (d) medication/diseases affecting cardiovascular system, muscle, or joints/bone (*n* = 1), (e) <100 Watt at ergometry (*n* = 0), (f) obesity (BMI > 35 kg/m^2^; *n* = 2), and (g) more than 2 weeks of absence during the interventional period (*n* = 3). After detailed presentation of the study protocol, sixteen subjects were unwilling to join the randomization procedure and quit the study. Thus, finally 81 subjects were randomly assigned (computer generated block randomization (*n* = 2–4), stratified for age only) to two subgroups: (a) high intensity (interval) training (HI(I)T) group and (b) waiting-control group/moderate intensity continuous exercise (MICE) group, respectively, after crossover (Figures 1 and 4).

### 2.3. Measurements

Each participant was tested by the same researcher and at the same time of day (±1 h). All assessments were determined in a blinded fashion. Researchers were not allowed to ask subjects about their exercise status (HIIT or waiting CG/MICE). Each subject was provided with standardized rules for behaving prior to the tests in order to prevent confounding effects on primary and secondary endpoints (i.e., time to exhaustion (TTE) during stepwise treadmill test, blood pressure, and body composition).

#### 2.3.1. Anthropometry

Height was determined with a stadiometer (Holtain, Crymych Dyfed, Great Britain). Weight, total, and regional body composition were determined using the bioimpedance technique (Inbody 230, Biospace, Seoul, Korea). Waist circumference was measured as the minimum circumference between the distal end of the rib cage and the top of the iliac crest along the midaxillary line.

#### 2.3.2. Metabolic Syndrome *Z*-Score

MetS-*Z*-Score was calculated according to the formula proposed by Johnson et al. [25] based on the NCEP-ATP III criteria of the MetS [26]. According to these criteria five risk factors constituted the MetS: (1) raised triglyceride (TriGly) levels (≥150 mg/dL),(2) reduced HDL-C (<40 mg/dL for males), (3) raised blood pressure (≥130/85 mmHG), (4) raised fasting plasma glucose (≥100 mg/dL), and (5) waist circumference (WC ≥ 102 cm for males).

Following Johnson et al. [25], the ATP-III cut-point for a male population and the corresponding baseline standard deviation (SD) of the entire RUSH-cohort were used for each parameter (i.e., HDL-C and triglycerides) of the individual data. In detail, the *Z*-Score was calculated using [(40 − HDL-C)/SD HDL-C] + [(TriGly − 180)/SD TriGly] + [(Glucose–100)/SD Glucose] + [(WC − 100)/SD WC] + [(Mean arterial pressure (MAP) − 100)/SD MAP].

#### 2.3.3. Blood Parameters

Blood was sampled from an antecubital vein in the morning (7:00–9:00 a.m.) after a 12 h overnight fast. Serum samples were centrifuged at 3000 RPM for 20 min. Total cholesterol, LDL-cholesterol, HDL-cholesterol, triglycerides, glucose, and uric acid (all: Olympus Diagnostica GmbH, Hamburg, Germany) were determined. During another visit, blood pressure was determined in a lying position after assessment of resting metabolic rate over 30 min, using an automatic oscillometric device (Bosco; Bosch, Jungingen, Germany). Subjects refrained from coffee and/or tea for at least 12 h before testing and with more than 12 h passing since the last relevant physical exertion or exercise session.

#### 2.3.4. Aerobic Capacity (VO_2_max) and Running Economy

During a stepwise treadmill test (3 min/1 km/h steps; 1° slope) up to a voluntary maximum, VO_2_ was continuously determined breath by breath using an Oxycon mobile open spirometric system (Viasys, Conshohocken, PA, USA). For more details the reader is kindly referred to another publication [27]. Energy consumption/total work calculation according to the American College of Sports Medicine Guideline [28] is based also on VO_2_ and RER-assessment considering the individual aerobic threshold.

Running economy (RE) was assessed for the last 30 sec of the second stage of the test (5:30 to 6:00 min), which was consistently kept below the anaerobic threshold [29].

### 2.4. Study Intervention

Figure 1 shows the design of the study intervention. For a detailed description of the intervention program the reader is referred to another publication [27]. Briefly, participants of the HI(I)T exercise group started on 16-week high intensity (running) exercise training in September 2011. After a short break around Christmas, participants of former control/waiting group started their 16-week moderate intensity continuous endurance exercise training in January 2012 and exercised until May 2012 (Figure 1). Each subject was provided with an individual training log that prescribed the intensity, volume, and frequency of the running exercise for the following 4 weeks. In both exercise groups, the weekly frequency of running exercise was progressively increased from 2 sessions/week at baseline to 3-4 sessions/week after week 8. With respect to exercise intensity the exercise protocols were based on individual prescriptions of the heart rate (HR) in different metabolic areas which were determined by stepwise treadmill test to a voluntary maximum and calculated based on the individual aerobic threshold concept (IAT: minimum lactate −2.0 mmol/L) suggested by Dickhuth et al. [30]. The “Schwelle” Software (Schwelle Version 3, Bayreuth, Germany) was used to properly determine the IAT and the IAT-HR. In addition, the validity of the calculated IAT-HR was tested at baseline and after 8 weeks by running 30 min at this specification. Based on the subjects' perceived exertion (“too high/too low”) IAT-HR was readjusted. In order to generate isocaloric condition and a comparable work load (in kcal) in both groups, running duration per exercise session was prescribed longer in the MICE group (57 ± 9 versus 53 ± 9 min/session) in order to compensate the higher intensity of the HI(I)T group [28].

Two sessions per week were consequently supervised by instructors who randomly checked exercise intensity by monitoring the HR watches (Polar RS 300, Kempele, Finland) which were provided for each participant.

#### 2.4.1. HI(I)T Protocol

**Figure 2:**
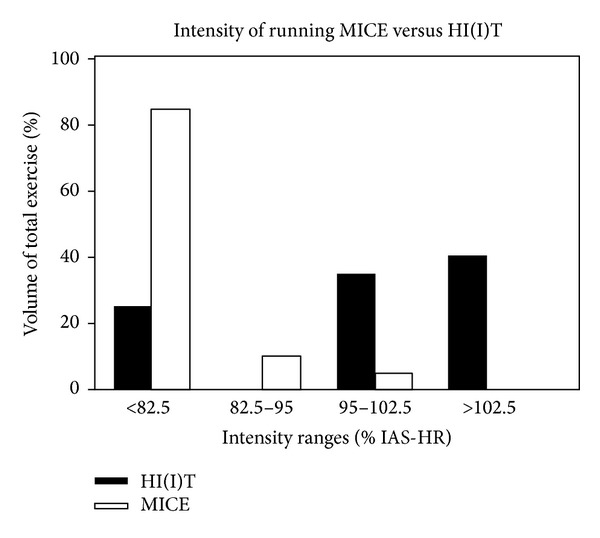
Exercise volume in different metabolic areas: HI(I)T versus MIC-running group. IAT: individual anaerobic threshold; HR: heart rate.

Figure 2 shows the rate of exercise volume in different metabolic areas for the HI(I)T and the MICE protocol. Briefly (core) exercise intensity during the intervention varied around 95%–110% of the IAT-HR (i.e., >85 to 97.5% HRmax) depending on the length of the interval (90 sec–12 min). Active rest periods of jogging or fast walking (1–3 min at 70–75% IAT-HR, *≈*65–70% HRmax) were prescribed between the intervals. Besides these intervals, continuous high intensity running sessions ranging from 25 to 45 min directly performed at the IAT (*≈*85% HRmax) were also part of the HI(I)T program. However 25% of total exercise volume of the HIIT group was performed at low exercise intensities (70–82.5% IAT-HR) during warm-up, cool-down, and the active rest periods (Figure 2).

#### 2.4.2. MICE Protocol

With slight exceptions (IAT-HR tests), exercise intensity of the MICE intervention was prescribed consistently low to moderate (70–82.5% IAT-HR) (Figure 2). Duration of (continuous) running exercise was progressively increased during the 16 weeks of intervention and ranged from 35 to 90 min per session.

### 2.5. Statistical Procedures

The sample size calculation was based on another study endpoint (“left ventricular end-diastolic volume”) that is not presented here. Based on the sample size of 40 subjects per group and a Type 1 error of 5%, the statistical power (1 − *β*) to detect a 5 ± 7.5% difference between the groups was 85%. All the subjects who took part in the follow-up measurements were included in the analysis (“finisher analysis”).

**Figure 3:**
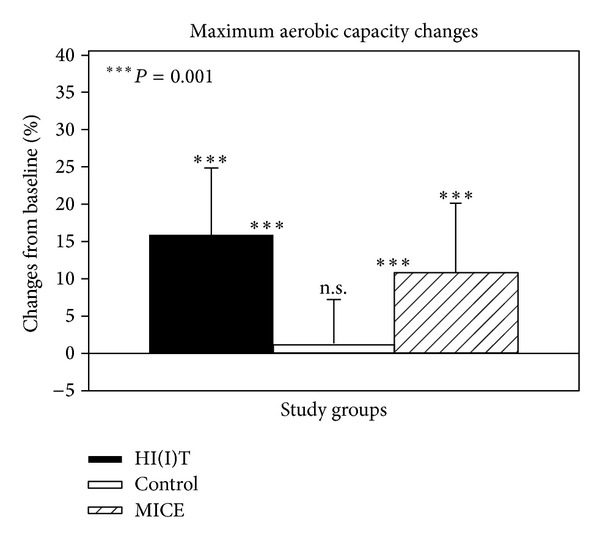
Percentage changes of relative VO_2_max in the RUSH study groups including the inactive CG. Asterisk (∗) on the upper end of the error bar indicates significant changes within the group and asterisk between the bars indicates significant group differences. n.s. = nonsignificant.

Baseline and follow-up data are reported as mean values and standard deviations. Changes between baseline and follow-up in HI(I)T and MICE were reported as absolute (Table 2) and percentage changes (text, Figure 3). In addition, mean differences (with 95% confidence intervals) between HI(I)T and MICE based on absolute changes are reported (Table 2). Differences of baseline characteristics were checked by one-way Anova. Distribution of the data was statistically and graphically checked. Nonnormally distributed parameters were log-transformed to obtain normally distributed data. Differences within groups were consistently analyzed by paired *t*-tests. Analyses of variance with repeated measurements consequently adjusted for baseline values were performed to check time group interactions. With respect to the comparison of all groups, post-hoc “Scheffé test” was also calculated (Figure 3). All tests were 2-tailed; statistical significance was accepted at *P* < .05. Effect sizes (ES) were calculated using Cohen's d. All statistical procedures were calculated with SPSS 21.0 (SPSS Inc., Chicago, IL) statistical procedures.

## 3. Results

Seven subjects of the HI(I)T (17.5%) and nine subjects of the MICE group were lost to follow-up (21%), while all the subjects of the CG (*n* = 41) were assessed. In this context, three subjects of the HI(I)T and four subjects of the MICE group quit the study due to injuries related to running exercise. One participant per group withdrew due to injuries related to the training protocol, two subjects of the HI(I)T and one subject of the MICE group due to injuries or diseases unrelated to exercise. Three subjects of the MICE and one subject of the HI(I)T group lost interest and cancelled their participation or did not start MICE after the control phase (*n* = 2).

Due to the comparable attendance rate of the HI(I)T (40.5 ± 5.4 sessions; 82.6 ± 11.1%) and MICE group (40.3 ± 5.9 sessions; 82.3 ± 12.0%) and the approach to compensate the higher exercise intensity of the HI(I)T group by higher exercise volume applied among the MICE group (2303 ± 352 min versus HI(I)T: 2092 ± 298 min; *P* = .012), total work was comparable between both groups (MICE: 30479 ± 6566 versus HI(I)T: 28966 ± 5228 kcal, *P* = .317).

**Figure 4:**
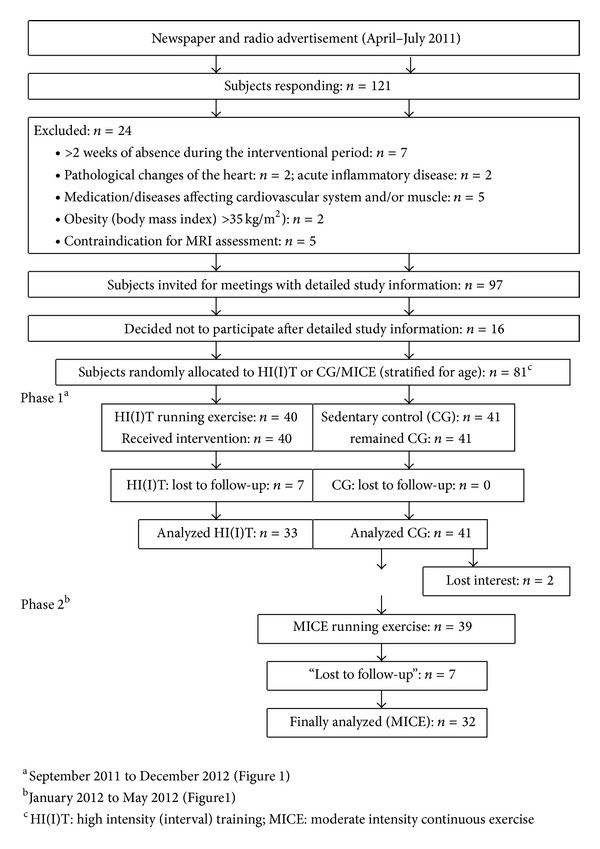
Flow chart of the RUSH study.

Table 1 gives baseline characteristics of the subjects. With respect to these and other confounding factors (i.e., diet, lifestyle, exercise, medications, or diseases) that may have impacted the present results, no relevant differences at baseline (Table 1) or changes from baseline to follow-up were observed during study phase 1 or 2 (see Figure 4).

Weight significantly decreased in both exercise groups (HI(I)T: −1.3 ± 2.3 kg, *P* = .004 versus MICE: −2.5 ± 2.4 kg, *P* = .001); however, the reduction was significantly higher (*P* = .046) among the MICE participants. Significant weight gain (1.2 ± 2.3 kg, *P* = .002), resulting in significant differences (*P* = .001) compared to MICE and HI(I)T, was observed for the CG. Lean body mass was maintained in the HI(I)T- (0.4 ± 2.1%, *P* = 0.381) and CG (0.7 ± 2.3%; *P* = 0.072) but significantly dropped in the MICE group (−1.1 ± 1.8%, *P* = 0.003). Body fat mass similarly (*P* = .261) decreased in both exercise groups (HI(I)T: −4.9 ± 9.0%, *P* = 0.010 versus MICE: −9.5 ± 10.4%, *P* < 0.001) but increased in the CG (3.4 ± 9.1%, *P* = .012).

### 3.1. Study Endpoints

At baseline MetS-*Z*-Score and number of MetS parameters of the HI(I)T group were significantly higher compared with MICE and control (Table 2).

Metabolic Syndrome score according to Johnson et al. [25] significantly improved (*P* = .001) in both exercise groups (HI(I)T: −2.06 ± 1.31 versus MICE: −1.60 ± 1.77). Although changes of the control group were also significant (−0.30 ± 0.75, *P* = .0.42) changes of both exercise groups were significantly more favorable (*P* = .001) (Table 2). At the same time, the number of MetS risk factors declined significantly (*P* = .001) in both exercise groups (HI(I)T: 21.9 ± 25.6% versus MICE: 43.8 ± 31.5%; *P* = .336) and was unchanged in the CG (1.3 ± 25.6, *P* = 0.729). Changes of both exercise groups for this parameter significantly differed (*P* = .001) from the CG (3.5 ± 17.9%, *P* = .432)

Thus the primary hypothesis that HIIT is significantly more effective compared with MICE to impact the Metabolic Syndrome (MetS) *Z*-Score and the number of criteria of the MetS clearly has to be rejected.

With respect to underlying mechanisms, Table 2 shows changes of parameters that constituted the MetS according to the NCEP ATP III MetS definition. Waist circumference was significantly reduced in both exercise groups (HI(I)T: −2.3 ± 2.7% versus MICE: −2.6 ± 2.9; *P* = .720), while a nonsignificant rise (*P* = .083) was observed for the CG (0.6 ± 2.3%; *P* = .001 compared with both exercise groups). At the same time, significant reductions of MAP (*P* = .001) were observed for both exercise groups (HI(I)T: −4.9 ± 4.0% versus MICE: −5.9 ± 4.2; *P* = .318) that significantly differed from the result of the CG (0.3 ± 4.4%; *P* = .611).

After adjusting for baseline values, no intergroup differences were determined for triglycerides that declined in all groups (HI(I)T: −19.7 ± 24.8%, *P* = .001 versus MICE: −4.6 ± 28.8%, *P* = .361 versus CG: −6.8 ± 33.9%, *P* = .208). HDL-C also changed favorably and reached significance level for all groups (HI(I)T: 8.8 ± 5.3%, *P* = .001 versus MICE: 2.3 ± 4.8%, *P* = .014 versus CG: 2.9 ± 4.3%, *P* = .001). However, changes between the HI(I)T and the MICE as well as between HI(I)T and CG were significant (*P* = .001), while no difference was observed between MICE and CG (*P* = .912). Furthermore, no group (HI(I)T: −1.4 ± 10.0% versus MICE: −0.8 ± 6.6% versus CG: −3.5 ± 16.7%) showed significant changes (*P* < .212) of fasting glucose. Lastly, no between group differences were determined for this parameter (*P* < .637).

(Relative) VO_2_max (mL∗min^−1^∗kg^−1^) significantly (*P* = .001) increased in both exercise groups (HI(I)T: 15.6 ± 9.3% versus MICE: 10.6 ± 9.6%) with no significant difference between HIIT and MICE (*P* = .121). Relative VO_2_max did not relevantly change in the CG (1.1 ± 6.4%, *P* = .263), thus, changes of both exercise groups significantly differed (*P* = .001) from the control. Of interest, nonweight adjusted absolute VO_2_max (mL/min^−1^) differed significantly (*P* = .002) between both exercise groups in favor of HIIT (14.7 ± 9.3%, *P* = .001 versus MICE: 7.9 ± 7.4%, *P* = .001); however due to the significantly more distinct reductions of weight (but not LBM) in MICE this finding disappeared after calculating relative VO_2_max changes (Figure 3).

Although these results make it difficult to generate a clear statement, for reasons discussed below, we also reject the secondary hypothesis that HIIT was significantly more effective to increase “cardiorespiratory fitness” compared with MICE.

## 4. Discussion

Summarizing the results, this study failed to detect a superiority of a HI(I)T compared with a MICE protocol on the Metabolic Syndrome (MetS) as a sensitive cluster of metabolic and cardiac risk factors even under the premise of comparable total “work” (…or “total energy consumption”) in both protocols (Table 2). However, compared with a nontraining control group both exercise protocols were highly effective for impacting the MetS, albeit by different mechanisms. While both protocols were similarly effective in reducing blood pressure and waist circumference, the effect of HIIT on blood lipids/lipoproteins was considerably higher compared with MICE. Three studies that either compared a high intensity interval [9, 21] or a high intensity continuous exercise protocol [25] with a MICE protocol while adjusting for energy consumption focused on the MetS cluster. In their pilot study with 22 subjects (52 ± 4 yr.) suffering from MetS according to WHO [31], Tjønna et al. [13] compared a HIIT (4 × 4 min at 90–95% HRmax intermitted by 3 min of active rest at 70% HRmax) with a traditional continuous running exercise training (47 min at 70% HRmax) performed 3times per week. After 16 weeks of exercise the HIIT subgroup showed a significantly more pronounced reduction of the number of subjects with MetS (HIIT: −46%, *P* < .05 versus MICE: −37% (*P* = .13) and the number of MetS criteria (−32%, *P* < .001 versus 12%, *P* < .05) compared with MICE. However, applying continuous running exercise over 6 months for overweight subjects with “mild-to-moderate dyslipidemia” (*n* = 86, 40–65 yr.) Johnson et al. [25] did not confirm this superiority of high intensity exercise (65–80% VO_2_peak; 128 min or 19 km/week) compared with MICE (40–55% VO_2_peak; 205 min or 19 km/week). In contrast, although nonsignificant, the authors reported more favorable changes of MetS-*Z*-Score and number of MetS criteria according to NCEP-ATP III among the MICE subgroup. However, with respect to the general effectiveness of these exercise protocols on MetS parameters, both MICE and HI(I)T significantly outperformed the corresponding control group(s). Earnest et al. [9] generally confirmed the data of Johnson et al. [25] in their comparative exercise trial with 42 overweighed 32–60-year-old males. After 6 weeks of exercise (3-4 × *≈*40 min/week with 50–70% VO_2_max) subjects were randomly allocated to further 6 weeks of continuous running exercise at 50–70% VO_2_max or a HIIT-protocol including up to 8 cycles of 2 min at 90–95% VO_2_max intermitted by 2 min of active recovery (50% VO_2_max). MetS-*Z*-Score according to the American Heart Association [32] and the number of MetS-risk factors (HIIT: −1.14 ± 1.15 versus MICE: −1.03 ± 1.68) significantly improved in both groups similarly. However, in a subgroup of persons at lower risk for insulin resistance (i.e., low HOMA-IR) only HIIT showed significant results. With respect to aerobic capacity/endurance performance both groups similarly and significantly improved (MICE: 3.1 versus HIIT: 2.9 mL∗min^−1^∗kg^−1^ or 2.8 versus 3.1 min during treadmill test). Of relevance, since body mass decreased in both groups (−1.3 versus −2.3 kg), absolute VO_2_max per se was not significantly affected (0.25 versus 0.13 L∗min^−1^) by the running protocols.

Revisiting the issue of whether some cardiometabolic MetS parameters are more sensitive to HIIT or to MICE, one might think that the large number of comparative trials, reviews, and meta-analysis that focus on this topic (e.g., [17, 33–37]) should allow a meaningful conclusion. However, in view of the large variety of confounders in these studies (e.g., sex, age, initial levels, health and fitness status, prescribed medication, and type of exercise) that may impact the effects of exercise on cardiometabolic risk factors, it is difficult to draw general conclusions. Also problematic for an adequate interpretation, most dose-response or comparative studies compared continuous (not interval) high intensity endurance protocols that did not reach intensities (far) above the aerobic threshold [38]. Some of these studies that classified “moderate” or “vigorous” intensity at the lower end of the ACSM classification [33] (i.e., 3–6 MetS for moderate and 6–8 for vigorous exercise intensity) prescribed “high intensity workouts” that hardly exceed the exercise intensity of the MICE group in this study [36]. Taking these limitations into account and simply summarizing the data of present comparative studies, we are unable to detect a consistent superiority of HIIT versus MICE protocols (or vice versa) on cardiometabolic risk factors related to the MetS, independent of health, overweight, and fitness status [9, 13, 17, 22, 23, 39–42], although there are indeed some studies that reported more positive effects on single MetS risk factors for MICE or HIIT (e.g., [23, 24, 43]).

With respect to “cardiorespiratory fitness”, a strong tendency for higher VO_2_max changes generated by the HIIT protocol of the present study was observed, which is mainly confirmed by comparative trials with untrained subjects with MetS or CHD/CVD [16, 17, 19, 20]. However, it is debatable whether higher VO_2_max changes actually represented higher changes of cardiorespiratory fitness. In fact, this and other studies (e.g., [9]) observed even slightly higher changes of endurance performance (speed_max⁡_, time to exhaustion) by their MICE protocols, although VO_2_max changes may be more distinct in the HI(I)T group. At first sight this result may appear contradictory, since VO_2_max is an important predictor of endurance capacity [44]. However, it is not the only determinant of this complex topic [45, 46]. In fact, changes of running economy, another central determinant of endurance capacity, were significantly higher in our MICE group, which may account for the similar changes of endurance capacity. Thus, generating a comparable effect on endurance performance per se (i.e., TTE) the mechanism of HIIT versus MICE widely differs with respect to maximization (VO_2_max) and economization (RE). Hence, although we think that VO_2_max may in general be a valid characteristic for cardiorespiratory fitness, a conclusion whether or not HIIT protocols are superior for impacting cardiorespiratory fitness should not be based solely on this parameter.

Some limitations and features of the RUSH study may complicate a direct comparison with most HIIT protocols cited above: (1) due to the study protocol the exercise groups were not evaluated in parallel but consecutively (Figure 1). Thus, seasonable changes of physical activity or diet may have impacted our results, although no corresponding changes were detected by the follow-up questionnaires. (2) The study protocol prescribed a combination of HIT and HI(I)T with intervals of 90 sec to 12 min but also continuous bouts (25–40 min) at the IAT. Although around or above the aerobic threshold, this approach differs from purebred “HI(I)T” protocols defined as repeated very short (<45 sec) or short (2–4 min) bouts of high to near maximum intensity exercise [47]. (3) Although on the majority overweight, only in 33% of the RUSH participants the MetS was prevalent. Correspondingly, the average numbers of MetS criteria per subject were rather low (2.0 ± 1.2), which may prevent a more distinct reduction of this parameter. Therefore the authors opt for the use of the MetS-*Z*-Score, a continuous score that is based on individual subjects (raw) data, which may be more appropriate to properly assess the effect of the study intervention [25]. (4) The drop-out rates in both exercise groups were rather high (*≈*20%). Independent of the exercise intensity, half of these subjects cited orthopedic problems for their withdrawal. Furthermore, a slight majority (HIIT: *n* = 16 versus MICE: *n* = 15) of the remaining males reported musculoskeletal problems and complaints longer than 7 days. These adverse effects may be related to the high body weight in this cohort and the abruptly increased mechanical impact due to the unaccustomed running exercise and the speedy progression of the running program. (5) With respect to exercise (intensity) compliance the authors did not analyze all the heart rate watches after the session but randomly selected 15–20 subjects per session (i.e., 50%) for this procedure.

(6) In order to adequately focus on the effect of exercise intensity, unlike most other studies with healthy untrained persons, comparable work load for both groups was prescribed. However, the authors are aware that time effectiveness is an important benefit of HIIT protocols that largely account for its attractiveness [48].

In summary, both protocols favorably impacted cardiometabolic risk factors; however the superiority of HIIT protocols for positively impacting MetS as a cluster of relevant metabolic and cardiac risk factors cannot be confirmed, at least for this cohort of untrained middle-aged males. Furthermore, although VO_2_max changes were significantly higher in the HI(I)T group, based on the slightly higher MICE effect on endurance capacity, we do not share the enthusiasm of other researchers in recommending pure HIIT protocols for increasing “cardiorespiratory fitness”. We would rather favor a skilled combination of higher and lower exercise intensity (and volume) that may optimally affect performance parameters related to both economy and maximization.

## 5. Conclusion

Addressing the strengths and limitations of HIIT protocols in the field of primary and secondary prevention, time efficiency along with effectiveness is a clear pro for HIIT, taking into account the sedentary lifestyle and low affinity to exercise of most middle-aged subjects [7]. At the same time, there is some evidence that HIIT was perceived as “more enjoyable” compared with the more monotone MICE [49] another pro that may increase exercise adherence. One may argue that the rather high intensity of HIIT may provoke cardiovascular complications during exercise at least in subjects with cardiovascular diseases. However, although the statistical power of this study may be still (*≈*176.000 exercise training hours at all) too low to detect differences between groups, a recent study of Rognmo et al. [12] listed extremely low rates of CHD events for both methods HIIT and MICE.

Thus, although we cannot confirm the general superiority of HIIT, with respect to efficiency and adherence, we strongly recommend HIIT as a reasonable component of endurance exercise protocols for prevention and rehabilitation of cardiometabolic diseases.

## Figures and Tables

**Table 1:** Baseline characteristics of the RUSH study groups. No significant differences between the groups were determined.

Variables	HI(I)T group	MICE group	CG
Age (years)	43.9 ± 5.0	42.9 ± 5.1	42.5 ± 5.6
Body height (centimeter)	181.1 ± 7.0	181.6 ± 5.3	181.7 ± 5.3
Body weight (kilogram)	91.5 ± 14.0	89.5 ± 12.3	89.8 ± 15.5
Overweight (BMI > 25.0 kg/m^2^) (*n*)	78%	73%	77%
Fat mass (%)^a^	25.5 ± 5.7	23.8 ± 6.0	23.9 ± 6.1
Physical activity (index)^b^	2.8 ± 1.4	2.6 ± 1.1	2.6 ± 1.1
Occupational working time (hours/week)	42.7 ± 8.5	44.8 ± 5.4	44.6 ± 7.4
VO_2_max (mL/min/kg^−1^)	35.9 ± 5.6	39.5 ± 5.5	37.9 ± 6.3
Total exercise volume (minutes/week)	28.8 ± 32.1	32.4 ± 37.3	32.6 ± 37.5
“Athletic” history (*n*)^c^	31	29	29
Energy intake (kilocalorie/day)	2595 ± 738	2737 ± 592	2580 ± 616
Fat/protein/carbohydrates (gram/day)	96/104/305	105/105/322	96/104/297

^a^As assessed by bioimpedance analysis; ^b^based on a scale from 1 (very low) to 7 (very high) according to a subjective assessment of professional, household, and recreational activities; ^c^formerly (13 ± 5 years ago) engaged in competitive sports with relevant demands on aerobic capacity (running, swimming, biking, triathlon, soccer, handball, and hockey); BMI: body mass index.

**Table 2:** Effects of high intensity (interval) training (HI(I)T) versus moderate intensity continuous exercise (MICE) on Metabolic Syndrome parameters. Intergroup differences were adjusted for baseline values.

	HI(I)T^a^	MICE^a^	Mean difference 95% CI	*P*	ES^b^(d)
Metabolic Syndrome index (*Z*-Score)					
Before	0.95 ± 3.36	−1.82 ± 2.53	—	.001	
After	−1.11 ± 3.46	−3.42 ± 2.88	—	—	
Difference	−2.06 ± 1.31^∗∗∗a^	−1.60 ± 1.77^∗∗∗a^	0.46 (1.07 to −0.22)	.291	0.29
Numbers of risk factors of the Metabolic Syndrome (*n* out of 5)					
Before	2.51 ± 1.29	1.60 ± 1.13	—	.005	
After	1.97 ± 1.20	0.90 ± 1.09	—	—	
Difference	−0.55 ± 0.62***	−0.70 ± 0.59***	0.15 (−0.16 to 0.46)	.336	0.25
Waist circumference (cm)					
Before	101.5 ± 9.0	98.0 ± 9.5	—	.129	
After	99.2 ± 9.2	95.4 ± 9.3	—	—	
Difference	−2.3 ± 2.8***	−2.6 ± 3.0***	0.26 (−1.18 to 1.70)	.720	0.10
Mean arterial pressure (MAP) (mm/Hg)					
Before	111.5 ± 10.1	104.7 ± 7.1	—	.003	
After	106.6 ± 8.9	98.8 ± 7.2	—	—	
Difference	−4.9 ± 4.0***	−5.9 ± 4.2***	1.03 (−1.01 to 3.06)	.318	0.24
Triglycerides (mg/dL)					
Before	166.3 ± 85.1	127.0 ± 55.2	—	.013	
After	146.5 ± 82.2	122.4 ± 59.4	—	—	
Difference	−19.7 ± 26.8***	−4.6 ± 28.8^n.s.^	15.1 (2.0 to 28.2)	.083	0.54
High density lipoprotein Cholesterol (mg/dL)					
Before	43.1 ± 12.1	50.4 ± 10.7	—	.001	
After	51.9 ± 12.7	52.7 ± 10.4	—	—	
Difference	8.8 ± 5.3***	2.3 ± 4.8*	6.46 (3.88 to 9.05)	.001	1.29
Glucose (mg/dL)					
Before	92.2 ± 11.3	89.6 ± 8.3	—	.258	
After	90.7 ± 7.6	88.8 ± 8.8	—	—	
Difference	−1.5 ± 10.0^n.s.^	−0.8 ± 6.6^n.s.^	0.76 (5.18 to −3.46)	.693	0.008

^a^Asterisk (∗) indicate changes within the group (*P < .05; **P < .01; ***P < .001).

^b^ES: effect size; 0.2: small; 0.5: moderate; 0.8: large.
